# Predicting effects of warming requires a whole-of-life cycle perspective: a case study in the alpine herb *Oreomyrrhis eriopoda*

**DOI:** 10.1093/conphys/coab023

**Published:** 2021-04-28

**Authors:** Annisa Satyanti, Toton Liantoro, Morgan Thomas, Teresa Neeman, Adrienne B Nicotra, Lydia K Guja

**Affiliations:** 1 Division Ecology and Evolution, Research School of Biology, The Australian National University, Robertson Building, Acton, ACT 2601, Australia; 2 Centre for Plant Conservation—Botanic Gardens, Indonesian Institute of Sciences, Jalan Ir. Haji Juanda, Bogor 16003, Indonesia; 3 National Seed Bank, Australian National Botanic Gardens, Parks Australia, Clunies Ross St, Acton, ACT 2601, Australia; 4 School of Earth, Environmental and Biological Sciences, Faculty of Science and Engineering, Queensland University of Technology, QLD 4067, Australia; 5 Statistical Consulting Unit, The Australian National University, Acton, ACT 2601, Australia; 6 Centre for Australian National Biodiversity Research, (a joint venture between the Parks Australia CSIRO), Clunies Ross St, Acton, ACT 2601, Australia

**Keywords:** Germination temperature, intraspecific trait variation, maternal effects, phenotypic plasticity, seed development temperature, transgenerational effects

## Abstract

Global warming is affecting plant phenology, growth and reproduction in complex ways and is particularly apparent in vulnerable alpine environments. Warming affects reproductive and vegetative traits, as well as phenology, but seldom do studies assess these traits in concert and across the whole of a plant’s life cycle, particularly in wild species. Thus, it is difficult to extrapolate from such effects to predictions about the persistence of species or their conservation and management. We assessed trait variation in response to warming in *Oreomyrrhis eriopoda*, an Australian native montane herb, in which populations vary in germination strategy (degree of dormancy) and growth characteristics as a function of ecological factors. Warming accelerated growth in the early stages of development, particularly for populations with non-dormant seed. The differences in growth disappeared at the transition to reproduction, when an accelerating effect on phenology emerged, to varying degrees depending on germination strategy. Overall, warming reduced flower and seed production and increased mortality, indicating a reduction in reproductive opportunities, particularly for populations with dormant seed. Developmental condition affected germination strategy of the next generation seed, leading to increased degree of dormancy and slowed germination rate. But there were no whole-scale shifts in strategy or total germination percent. Following through the life cycle reveals that warming will have some potentially positive effects (early growth rates) and some negative effects (reduced reproductive output). Ultimately, warming impacts will depend on how those effects play out in the field: early establishment and an accelerated trajectory to seed maturity may offset the tradeoff with overall seed production. Small differences among germination strategies likewise may cascade to larger effects, with important implications for persistence of species in the alpine landscape. Thus, to understand and manage the response of wild species to warming takes a whole-of-life perspective and attention to ecologically significant patterns of within-species variation.

## Introduction

Addressing the impact of global warming on plants requires an understanding of multiple traits across plant life stages, not only within but also across generations. Warming, as associated with climate change, has a strong effect on phenotypic traits throughout a plant’s life cycle. Warmer temperatures generally lead to increased biomass and vegetative growth (Debouk *et al.*, 2015, King *et al.*, 1999, Lin *et al.*, 2010, Walker *et al.*, 2006), reduced flower numbers and seed production (Hedhly *et al.*, 2009, Liu *et al.*, 2012), increased mortality at both seedling and adult stages (Allen *et al.*, 2010, Hovenden *et al.*, 2008, Milbau *et al.*, 2017) and delays and decreases in seedling emergence (Cochrane *et al.*, 2015a) and potentially can lead to local extirpation of the species (Panetta *et al.*, 2018). Phenology, the timing of biological events—e.g. bud burst, flowering, seed maturation and senescence—has emerged as the characteristic that is most sensitive to warming (e.g. Arft *et al.*, 1999, Cleland *et al.*, 2007, Farnsworth *et al.*, 1995, Körner and Basler, 2010, Menzel *et al.*, 2006, Moore and Lauenroth, 2017, Munson and Sher, 2015, Peñuelas and Filella, 2001, Rathcke and Lacey, 1985, Root *et al.*, 2003, Sherry *et al.*, 2007, Walther *et al.*, 2002).

Warming responses of vegetative growth, reproductive output and phenological traits (including senescence) should not, however, be viewed in isolation (Hoffmann and Sgrò, 2011, Leblans *et al.*, 2017, Merilä and Hendry, 2014, Nicotra *et al.*, 2010). Rather, variation in trait values and timing of phenological events determines subsequent interactions of the individual with the environment and because of this co-dependence, the responses to environmental change are likely to be quite complex. For example, germination timing (spring versus autumn), mainly controlled by the degree of seed dormancy and germination requirements, determines the seasonal conditions and duration of seedling exposure as well as the conditions in which subsequent life stages (flowering time and lifespan) occur (Donohue, 2009, Lu *et al.*, 2016). Time to flowering, likewise, could determine the season of seed maturity with implications for alteration of the degree of seed dormancy and germination timing, and the germination timing could in turn determine whether the offspring will become annual or biennial (Galloway, 2005, Galloway and Etterson, 2007). Thus, a shift in the timing of one phenological event can have cascading effects for the subsequent life events. However, these shifts do not necessarily all occur in concert, for example, early flowering in response to warming is not always followed by a change in timing of cessation of flowering and thus may result in the expansion or contraction of the flowering period (CaraDonna *et al.*, 2014).

Warming effects may also be highly species or even population specific. For example, prairie species that have early flowering (spring to mid-summer) start to flower even earlier, and conversely, late-flowering species (mid-summer to autumn), delay their flowering when exposed to warming treatments (Dunnell and Travers, 2011, Sherry et al., 2007). In the sub-arctic herb *Cerastium fontanum*, plants from warmer microsites (populations) flower earlier than those from colder microsites, but when grown in much warmer common gardens, plants from warmer microsites start to flower later than plants originating from colder microsites (Valdés *et al.*, 2019). In the alpine herb *Aciphylla glacialis*, seedlings growing under a passive field warming treatment (an open top chamber) exhibit higher mortality (but surviving individuals grow faster) than those under ambient conditions (Geange *et al.*, 2020), regardless of the population source (Briceño *et al.*, 2014). Species response to warming may also be dictated by functional traits. Between geographically co-occurring species, the seedlings of fast-growing *Banksia coccinea*, characterized by higher specific leaf area (SLA) and leaf growth rate, can maintain growth at high temperatures better than its slow-growing congeneric *Banksia baxteri* (Cochrane et al., 2015a). Within a plant’s life cycle, response to elevated temperatures can also be trait specific. For example, in *Arabidopsis thaliana*, warming advances flowering time and accelerates vegetative development and fruit production but not plant mortality (Springate and Kover, 2014). So far, the studies on plant responses to climate change often focus only on particular suites of traits (e.g. leaf traits or flowering phenology) and only at a certain life stage (mostly adult reproductive), which may lead to incomplete assessments and failure to predict the cascading effects of warming on species’ persistence, and thus community composition and ecosystem functions and services.

While research on the adaptive capacity of plants under future climate is increasing, we still lack understanding of plant plasticity and the capacity to evolve when challenged by new environmental conditions, especially for germination and the seedling stage (Parmesan and Hanley, 2015). Studies across ontogeny that span whole life events, and multiple generations, are also lacking. Maternal conditions may have carry-over effects across generations and could determine the life history of the progeny (Donohue, 1998, Donohue, 2009). When these maternal effects increase offspring fitness, this transgenerational plasticity may be adaptive (Galloway, 2001a, Galloway, 2001b, Galloway, 2005, Galloway and Etterson, 2007, Herman and Sultan, 2011). For example, warmer temperatures during seed development generally reduce the degree of physiological seed dormancy (Bernareggi *et al.*, 2016, Gutterman, 2000, Hoyle *et al.*, 2008, Huang *et al.*, 2018), enabling individuals to start growing and to reach reproductive stage earlier in the season and thus have a longer duration for seed production and potentially produce more seeds (Donohue *et al.*, 2010, Roach and Wulff, 1987). Climate-induced variation in seed dormancy status, in turn, may shift timing of germination or variance therein, providing a buffer against disturbances that may be a bet-hedging strategy. Changes in dormancy may also affect seedling growth rate and establishment (Satyanti *et al.*, 2019) or physiological traits such as water use efficiency, reproductive phenology and senescence (Kimball *et al.*, 2010, Kimball *et al.*, 2011). Understanding the extent to which germination strategy, particularly the degree of dormancy and how it is lost over time, could change with warming and whether maternal temperature interacts with offspring germination requirements to affect germination success will be valuable for predicting species persistence in the face of a changing climate.

Alpine regions are recognized as one of the most vulnerable ecosystems under warming climates—impacts are forecast to be pronounced and detectable earlier than in other biomes (Grabherr *et al.*, 2010). The Australian alpine region and the services it provides are considered to be particularly vulnerable to global change, due to its small vertical range, limited extent and the relative isolation of high-elevation habitats (Hennessy *et al.*, 2007, La Sorte and Jetz, 2010, Laurance *et al.*, 2011, Reisinger *et al.*, 2014, Steffen *et al.*, 2009). Since upward migration will be impeded by habitat fragmentation and limited by the availability of suitable sites, alpine plant species may be particularly dependent on tolerance, plasticity and adaptation through modification of physiological processes at seeds and seedling life stages. Thus, understanding the impacts of warming on the life cycle and fecundity of alpine herbs will be important to predicting long-term impact of warming and to informing management and conservation practices.

Alpine plants that are adapted to low temperature and a short growing season are particularly responsive to warming (Anadon-Rosell *et al.*, 2014, Arft *et al.*, 1999, Bjorkman *et al.*, 2015, Cao *et al.*, 2016, Geange *et al.*, 2017, Geange *et al.*, 2020, Kudernatsch *et al.*, 2008, Kudo and Suzuki, 2003, Oberbauer *et al.*, 2013). Perhaps paradoxically, seeds of alpine plants generally require relatively high soil temperature to trigger germination (Hoyle *et al.*, 2013, Schütz, 2000, Schütz and Milberg, 1997), and hence, warming could have a positive effect on recruitment via seed. In strongly seasonal and unpredictable environments, plants often evolve specific seed dormancy strategies that lead to divergence in germination strategy among species (Hoyle *et al.*, 2015, Satyanti *et al.*, 2019, Willis *et al.*, 2014). For example, alpine species often exhibit physiological and/or morphophysiological dormancy (Fernandez-Pascual *et al.*, 2021, Schwienbacher *et al.*, 2011, Tudela-Isanta *et al.*, 2018). Variation in germination strategy across alpine species is common (Hoyle *et al.*, 2015, Körner, 2003, Satyanti, 2018), but more strikingly, intraspecific variation in germination strategy with environment and elevation is also documented (e.g. Hoyle *et al.*, 2015, Satyanti *et al.*, 2019, Vidigal *et al.*, 2016, Wagner and Simons, 2009). Such variation in germination strategy may help to facilitate the regeneration and survival of plants in short growing seasons and highly variable environments (Satyanti et al., 2019).

*Oreomyrrhis eriopoda* (Apiaceae) is a native rosette-forming herb from the Australian Alps that exhibits varying degrees of physiological dormancy that can be described as four germination strategies: immediate, staggered, postponed and postponed deep (Satyanti *et al.*, 2019). Populations with an immediate strategy produce non-dormant seed and thus ‘autumn seedlings’. Populations that produce dormant seeds (postponed strategy) produce ‘spring seedlings’. Populations were categorized as postponed-deep germinate (usually in spring) after exposure to multiple cycles of winter conditions. Populations that exhibit the staggered strategy produce both non-dormant and postponed seeds in the same accession and thus both ‘autumn’ and ‘spring seedlings’ occur (e.g. Hoyle *et al.*, 2015, Satyanti *et al.*, 2019). The among-population variation in germination strategy in *O. eriopoda* leads to substantial differentiation of seedling growth among and within populations; in staggered populations, autumn seedlings grow faster than spring seedlings (Satyanti *et al.*, 2019).

So far, we know little about the extent of intraspecific germination strategy variation in plant responses to warming, particularly, how potential impacts on growth and to phenology affect reproductive capacity and fitness and thus will scale to persistence in the face of rapid climate change. Using *O. eriopoda* as a case study, we aim to understand the effect of warming across all life stages (vegetative growth, reproductive output, phenology and the germination traits of the seed produced) and to explore whether populations of different germination strategies show variation in those warming responses in the current and subsequent generation. We hypothesized that (i) warming would interact with germination strategies and overall would enhance growth at the cost of flower and fruit production, and likely with an increase in plant mortality; (ii) plant responses to soil temperature would depend on germination strategy; and (iii) germination strategy of the offspring will be affected by warming of the maternal environment.

## Materials and methods

### Propagation

Accessions (population level seed samples) of *O. eriopoda* were sourced from the National Seed Bank of the Australian National Botanic Gardens Canberra, The Australian Botanic Gardens Mount Annan, The Royal Tasmanian Botanical Gardens and the Royal Botanic Gardens Victoria and germinated as described in Satyanti *et al.* (2019). We included seedlings from 16 populations (Fig. S1 and Table S1) that represented the range of germination strategies expressed in this species. We aimed for a balanced representation of germination strategies based on early assay results; however, there were many populations with postponed germination so we included more of those to be representative. Consequently, we had three populations with an immediate germination strategy, three with a staggered strategy, seven postponed and three with a postponed-deep germination strategy (Table S1). Seeds from each population were germinated in two batches so that germination of early (autumn) and late (spring) seedlings coincided and seedling age was consistent regardless of germination strategy (Appendix S1). This design addressed the potentially confounding factors of age, size and starting time of the experiment and allowed us to manage these factors within the logistical constraints of a blocked design (Lu *et al.*, 2016).

Seeds germinating on agar were transplanted within 1–2 weeks to soil media in pots and grown under temperatures conducive to growth. Seedlings were grown until they were 18–20 weeks old when they had 8 ± 3.6 (SD) leaves, and we inferred that the establishment stage was complete (Appendix S1). As *O. eriopoda* is slow growing, this establishment step was important to ensure that plants across all populations were large enough to withstand transplant stress and thus to avoid confounding soil warming effects.

### Experimental design

Soil temperatures are particularly important for alpine species because given their low stature, soil temperatures determine the microclimate to which plants are exposed more than the air temperatures do (Körner, 2003, Reinhardt and Odland, 2012, Scherrer *et al.*, 2011, Wolkovich *et al.*, 2012). We therefore focused our study on the effect of soil warming on plant traits. Because of the logistical challenge of assessing whole of life temperature effects in the field, these experiments were conducted in glasshouses under controlled temperature conditions.

For each population, 10 individuals (one per pot) were randomly allocated to each of the two soil warming treatments. We assessed initial seedling size and confirmed that there were no *a-priori* differences in the size of plants between ambient and warm soil treatments (linear mixed model with germination strategy as a random term; *P* = 0.286 and 0.52 for leaf number and leaf length, respectively). For the staggered germination strategy, both early (autumn) and late (spring) seedlings were placed in the respective blocks; thus, these populations had 20 individuals/soil warming treatments. One representative population of each germination strategy was assigned to each of the four blocks. The imbalance of populations for each germination strategy was not an impediment for the analyses. There were five populations for which we did not have 20 seedlings and for these we assigned half of the available number to each warming temperature. Extra plants were placed in the empty spaces on the bench to maintain homogeneous spacing across the experiment but were not included in analyses (number in grey, Fig. S1).

**Figure. 1:**
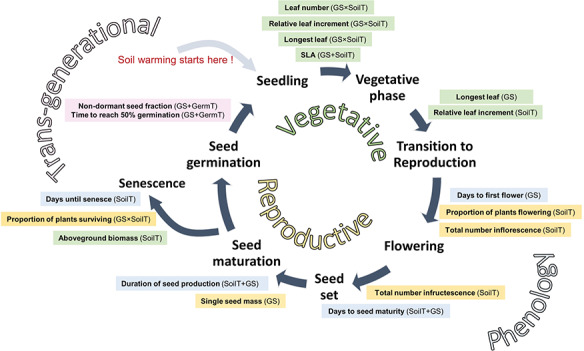
The life cycle of *Oreomyrrhis eriopoda* from early vegetative growth to reproduction and senescence. Of 19 traits measured, 16 traits were influenced by experimental factors: germination strategy (GS), development temperature (SoilT) or their interactions (GS × SoilT), at some stage. Transgenerational seed traits were only affected by germination temperature (GermT) and not maternal SoilT. Box colours indicate traits: vegetative (pale green), reproductive (pale yellow), phenology (pale blue) and the trans-generational effect on seed germination (pink). Significant fixed terms are given in brackets; detailed results are presented in Table 1, Table 2, Figs 2 to 4 and Tables S2 and S3.

Warm and ambient benches were set up in a glasshouse with air temperature set to follow the seasonal changes and natural photoperiod (Fig. S2). The targeted air temperature day/night sequence was 20/10°C (autumn), 5/5°C (winter), 20/10°C (spring), 25/15°C (summer) and finally 20/10°C (autumn). Soil warming was achieved by placing a heating mat (Electronic Foil Panel with Thermostat, ADLOHEAT, Victoria) on a given bench set to be continuously ~ 5°C warmer than the set glasshouse air temperature throughout the experimental period, including winter. The 5°C soil temperature increase is based on Australian alpine mean air temperature predictions for 2050, i.e. an increase of +0.6 to +2.9°C (Hennessy *et al.*, 2003) and that the maximum soil temperatures in Australia are to increase by almost double that of air temperature by the end of 21st century (Ooi *et al.*, 2009). The 5°C, thus, falls between the predicted +1.2 to 5.8°C soil temperature increase by 2050. The ambient treatment was located on benches that had mats but no heat, paired one each with the four heated benches. A frame of 6 mm-thick PVC sheet was placed around each bench, 17 cm above the mat, and a 5 cm-thick sheet of polystyrene foam was placed on the top of this to insulate the soil. Square openings in the polystyrene matched the pot size and held these in place in the frame. Temperature at plant level (15 cm above bench; Fig. 1) and soil (2, 8 and 14 cm below surface) was monitored during the course of the experiment with i-Button data loggers (Thermochron DS1921G, Temperature Technology, Adelaide) in each block (32 in total, Fig. 1). We analysed the temperatures at plant and soil level to determine efficacy of the design using analysis of variance and found significant warming differences for each of the soil and air depths/height. The temperature difference between ambient and warm soil at 8 cm below the surface (where most roots were located) was approximately 6°C during the day and 9°C during the night (Fig. 1), in agreement with climate patterns which show that night-time temperatures have increased more than day-time temperatures (Donat and Alexander, 2012, Easterling *et al.*, 1997). With warmer air temperature and reduced snow cover, the soil in the Australian Alps becomes warmer, evidenced by a snow removal experiment (Slatyer, 2016). Thus, we also increased the soil temperature during winter for the warmer soil scenario. In summer, soil was warmed to ~ 35°C, which is realistic for the Australian mountains where bare dark soils can easily exceed 45°C on sunny days and the difference to the nearby vegetated soils can be over 30°C (Slatyer, 2016).

The irrigation system was set to keep plants adequately watered. We used an automatic Water-Pro vapour pressure deficit (VPD) to provide an automatic watering system (MicroGrow Green House Systems, Temecula, California), with each plant being watered individually by a dripper at soil level. Drippers were calibrated to a standard flow rate that was checked at the beginning of the experiment. Watering events were triggered when pre-set VPD targets were reached. Plants received ~90 ml per watering, which was enough to saturate the soil at the start of the experiment. As the plants grew, we adjusted the VPD point, based on the two sensors for ambient and warm soils, so that the drippers delivered water sufficiently more frequently to the plants in the warmed treatment to ensure adequate water delivery and equivalent water status.

### Trait measurement

Leaf number and the length of the longest leaf were recorded for all plants at the start of the experiment and at the end of autumn (day 38). Those traits were recorded for a subset of plants (four individuals per soil temperature and population) in early spring (day 124). Leaf increment rate at the early vegetative stage was calculated from the difference of leaf number at the end of autumn from the start divided by 38 (number of days from planting). Leaf increment at the transition to reproductive stage was the difference between total leaf number at day 38 and day 124, divided by 86 (number of days between measurement).

SLA is an indicator of resource allocation and ecological strategy. We expected that plants growing faster in warmed conditions would have higher SLA. SLA was measured for every individual at the start of winter (day 52). SLA was measured by acquiring the youngest fully expanded leaf for each individual. The leaf was then scanned on a flatbed scanner, dried at 60°C for 72 h and weighed. The SLA was calculated as area/weight (cm^2^g^−1^).

In early spring, a random sample (four plants, even numbered individuals, per population per soil treatment) was photographed with a reference scale over a white Styrofoam board to determine total canopy area. Each plant image was then converted to an 8-bit graphic file. The threshold was adjusted so that only the actual canopy area was detected for selection, and using the known distance of the reference scale we calculated the plant area. The image analysis of the canopy area was performed in ImageJ (Schneider *et al.*, 2012).

Plants were monitored every 1–2 days for phenology. The date at which the first inflorescence with closed buds emerged from the plant base was recorded as first flowering. The total number of inflorescences and infructescence was determined for each plant. Further, we measured individual seed mass for five individuals per population per soil treatment by weighing three replicates of 25 seeds (Fig. S1). The date of collection of the last infructescence was recorded as the end of seed dispersal, and the time between the first and the last seed harvest was defined as the duration of seed production. Some plants naturally senesced over the course of the experiment, and the date of senescence (all leaves browned, no new leaves emerging) was recorded. At the end of the experiment, aboveground biomass was harvested for all plants, dried at 60°C to constant weight and weighed. Plants that died before harvest were sampled for biomass within a week of senescence.

### Transgenerational effect on germination traits

To minimize potential genetic influences that may have occurred from cross-pollination of populations, populations were separated by organza fabric sheeting supported by a plastic frame. Infructescences were collected from plants at the time of natural dispersal and stored separately at the plant level. Infructescences were kept in paper bags and stored in drying room (15% RH and 15°C) for approximately four months before cleaned and used in the germination assay.

Seeds from parent plants of three germination strategies, immediate, staggered and postponed, were used to test whether there was an effect of soil temperature during development on the germination strategy and response to germination temperature of progeny. Ten populations were selected to represent the three germination strategies, three immediate, three staggered and four postponed strategies (Fig. S1). Five fruiting individuals were randomly selected from each population and soil temperature combination. From each plant, 25 seeds that had been produced in the peak seed production period were sown on each of two Petri dishes and randomly allocated to one of two germination chambers set at 25/15°C and 30/20°C 12/12 h photoperiod for 9 weeks. Subsequently, the seeds were transferred to 5°C for 8 weeks and then returned for 17 weeks to the same temperature regimes they were initially allocated to (25/15°C or 30/20°C). In total we had 100 Petri dishes each containing 25 seeds in each of the temperature chambers for a total of 200 dishes. Germination was scored weekly as the seeds moved through the temperature regimes (beginning week 1 until week 34), to develop germination curves for each population and warming treatment. At the end of the experiment, a cut test was performed to determine whether ungerminated seeds were empty, dormant or dead. Each of the two chambers consisted of five blocks (shelves). For a given population, one maternal plant was represented in each block.

### Statistical analyses

Mixed models were selected for the analysis of plant traits. Models included terms for germination strategy and soil temperature and the interaction thereof as fixed factors, and populations, nested within blocks, were assigned as random factors. Some exceptions were made in the random model where either block or population was used as the random factor because inclusion of population nested in block resulted in convergence failure (see Table S2). Vegetative and reproductive traits that were discreet (number of leaves, number of inflorescences, number of inflorescences, day to flower, day to seed dispersal, seeding duration and day to senesce) were analysed using generalized linear mixed models (GLMM), setting the distribution family as Poisson and the link function as natural logarithm (Bolker *et al.*, 2009). Leaf number (but not length of the longest leaf) at the start of the experiment significantly varied across germination strategies (Table S2). Hence, we used leaf number at the start of the experiment as a covariate for corresponding traits, i.e. leaf number, leaf increment rate, SLA, plant area and aboveground biomass. For proportion data (survival and proportion of plants producing seed), GLMM were used with the distribution family as binomial, the link function as logit and the dispersion parameter set to be estimate. Responses that were continuous (longest leaf, leaf increment rate, individual seed mass, SLA and aboveground biomass) were analysed with linear mixed models (restricted maximum likelihood, REML). Leaf increment rates, plant area, seed mass and aboveground biomass were log transformed prior to fitting to REML (Table S2).

Repeated measures analyses were run for the leaf number and leaf length using germination strategy, soil temperature and measurement time as fixed factors. Population nested in block was used as the random model and leaf number at the start of the experiment as covariates for the analysis of the leaf number. However, the results were the same as when we performed the analyses for each measurement time point, and thus, we present the results from the two measurement points as they provide clearer visual inference.

To assess transgenerational effects on germination strategy, we analysed the final germination, non-dormant seed fraction (germination before spring) and time to reach 50% germination of the F1 seeds. The final germination and non-dormant seed fraction were analysed using GLMM, assigning germination temperature, soil temperature and germination strategy as the fixed factors, population and individual plants nested on the incubator shelf were set as the random factor, and we set the distribution family as binomial and the link function as logit. Time to reach 50% germination was derived by examination of cumulative germination for each dish to the closest 0.25 week and treated as a continuous variable and analysed using linear mixed models (REML) with fixed and random factors as for the non-dormant fraction analyses. GLMM and REML were performed in Genstat 19th Edition.

## Results

The effect of warming was pronounced over the course of the *O. eriopoda* life cycle, but patterns of effects varied among traits (Fig. 1). We measured 19 functional traits important to establishment, growth and fitness (Table 1) across three different life history stages (vegetative, reproductive and transgenerational) and analysed for significant effects of warming, germination strategy and interactions thereof. Sixteen of the 19 variables showed significant responses, i.e. either the interaction or at least one of the two main variables yielded significant effects (Table 1, 2).

**Table 1:** Significance values for analyses of vegetative, reproductive and phenology traits; full details in Supplementary Table S2. Mixed models were used in which germination strategy (GS), i.e. immediate (I), staggered (S), postponed (P) and postponed deep (PD), and soil temperature (SoilT), i.e. ambient (A) and warm (W), were assigned as the fixed factors and population nested in block as random factor. Where appropriate, leaf number at the start of the experiment was added as a covariate in the analysis in addition to block/population (‡)

Response	GS		SoilT		GS × SoilT	
Early vegetative stage						
Leaf number ‡	0.063		<0.001	A < W	<0.001	I (A < W), PD(A < W)
Relative leaf increment (leaf per day) ‡	0.063		<0.001	A < W	<0.001	I(A < W), PD(A < W)
Longest leaf	<0.001	I < S < PD < P	0.626		0.043	I(A > W)
Specific leaf area (cm^2^g^−1^)	0.041	P < (I, S, PD)	<0.001	A < W	0.903	
Transition to reproductive stage						
Leaf number ‡	0.208		0.363		0.533	
Relative leaf increment (leaf per day) ‡	0.417		0.004	A > W	0.179	
Longest leaf	0.043	I < (S, P, PD)	0.793		0.963	
Canopy area (cm^2^) ‡	0.458		0.875		0.333	
Reproduction stage						
Proportion of plants flowering	0.189		0.028	A > W	0.079	
Total number of inflorescences	0.143		<0.001	A > W	0.094	
Total number of infructescence	0.260		<0.001	A > W	0.094	
Single seed mass	<0.001	I < (S, PD) < P	0.527		0.174	
Phenology						
Days to first flower	<0.001	I < (S, P, PD)	0.645		0.238	
Days to seed maturity from flowering	<0.001	I > (S, P, PD)	0.002	A > W	0.139	
Duration of seed production	<0.001	I > (S, P, PD)	0.007	A > W	0.312	
Days to plant death from planting	0.614		<0.001	A > W	0.091	
Final survival and aboveground biomass						
Proportion of plants surviving	0.214		0.008	A > W	0.008	I(A > W), S(A > W), PD(A > W)
Aboveground biomass ‡	0.695		0.019	A > W	0.399	

**Table 2:** Significance values of transgenerational effects on germination strategy. Germination strategy (GS), maternal soil temperature (SoilT) and germination temperature (GermT) were assigned as fixed factors, with individual nested in population and shelf blocking as random factor. Generalized linear mixed models were used to analyse the proportion of non-dormant seed fraction and final germination. A linear mixed model was used to analyse the time to reach 50% of germination. The germination strategy (GS), abbreviated as immediate (I), staggered (S), postponed (P) and postponed deep (PD), and soil temperature (SoilT), i.e. ambient (A) and warm (W), were assigned as the fixed factors and population nested in block as random factor. Details in Table S3

Transgenerational seed germination responses	GS		SoilT	GermT		GS × SoilT	GS × GermT	SoilT×GermT	GS × SoilT×GermT
Non-dormant seed fraction	0.012	I > S > P	0.347	<0.001	A > W	0.318	0.131	0.308	0.425
Time to reach 50% of seed germination	<0.001	I < (S = P)	0.461	<0.001	A < W	0.227	0.192	0.135	0.753
Final germination	0.092		0.849	0.067		0.603	0.45	0.342	0.58

**Figure. 2:**
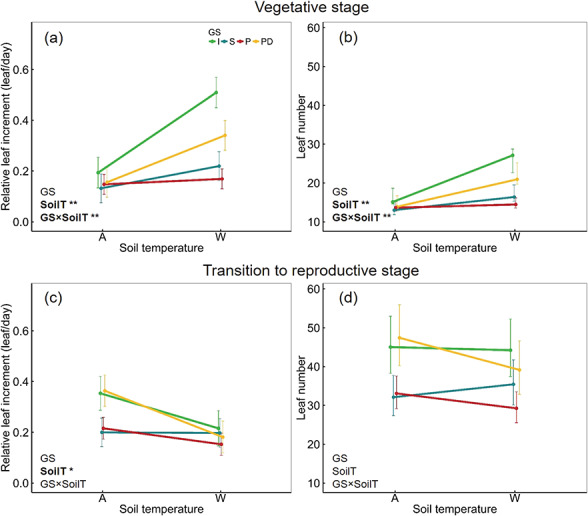
Vegetative growth under developmental soil temperature (SoilT, x-axis) across germination strategies (GS) indicated by the mean ± SE: (a) relative leaf increment and (b) absolute leaf number at vegetative stage, (c) relative leaf increment and (d) absolute leaf number at the transition to reproductive stage. Significant terms are indicated by the bolded term and asterisk; * = significant at *P* < 0.05, ** = significant at *P* < 0.001. Line colour represents germination strategy (GS): green (I: immediate), blue (S: staggered), red (P: postponed), yellow (PD: postponed deep).

## Vegetative growth

Soil warming significantly increased early vegetative growth for some germination strategies (Table 1). Soil warming significantly increased the number of leaves and the leaf increment (leaf number per day) in the immediate and postponed-deep germination strategies, but not the staggered or postponed strategy (Fig. 2a and b). Leaf size decreased under warmer conditions only in the immediate germination strategy, while for other strategies leaf number between ambient and warmer soil was constant. SLA differed across strategies and warming increased SLA regardless of the germination strategies (Table 1).

The effect of warming and germination strategies on vegetative growth diminished at the transition from vegetative to reproductive stage when many individuals had just started to flower. Rosette size indicated by the canopy area and leaf number were not affected by warming and germination strategy (Table 1). Interestingly, at the transition to reproductive stage, warming was associated with a reduction instead of an increase in leaf increment regardless of germination strategy—contrary to the response shown during the early vegetative state (Fig. 2, Table 1). Thereby, despite the positive effect of warming on vegetative traits at the early stage, final aboveground biomass of plants growing under warming treatment was significantly lower than that of ambient plants (Fig. 1, Table 1).

## Reproductive output

**Figure. 3:**
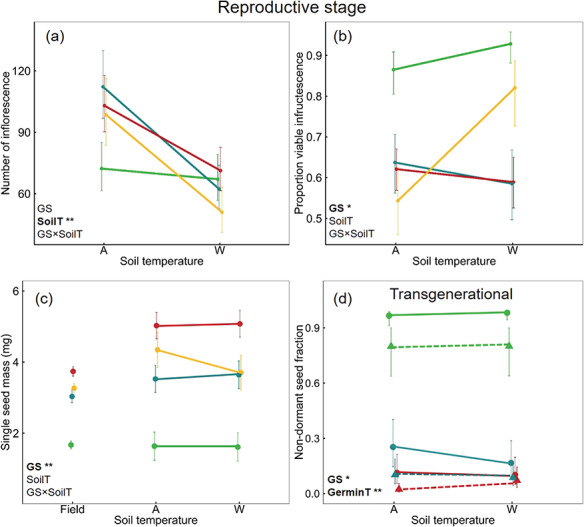
The effect of developmental soil temperature (SoilT, x-axis) and germination strategy (GS) during the reproductive stage on (a) number of inflorescences, (b) total number of infructescences, (c) the mass of a single seed. (d) shows the non-dormant seed fraction (autumn germination) of the subsequent generation contrasting seeds germinated at 25/15°C (●—full line) with those from 30/20°C (▲—dashed line). Values are mean ± SE. Line colours represent GS: green (I: immediate), blue (S: staggered), red (P: postponed), yellow (PD: postponed deep). Significance values are indicated by bolded terms and asterisk. * = significant at *P* < 0.05, ** = significant at *P* < 0.001.

**Figure. 4:**
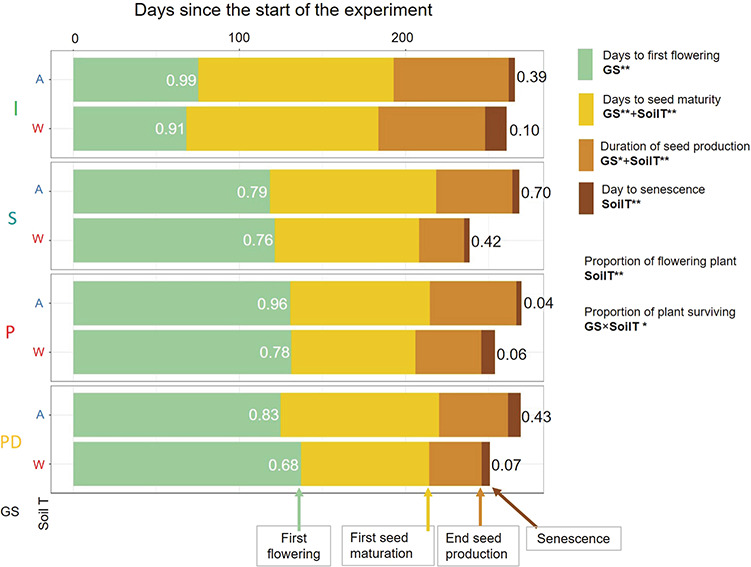
The reproductive phenology and senescence progression are indicated by the different bar colours: day to first flower emergence (green), day to first seed maturation from flowering (gold), the duration of seed production (first to last seed maturation, light brown) and senescence (dark brown). Numbers in white font (left) indicate the proportion of plants flowering and in black font (right) indicate the proportion of plants surviving. In the legend, bolded terms, i.e. developmental soil temperature (SoilT) and germination strategy (GS), and asterisks indicate significance (Table 1, Table S2).

The effect of soil warming on reproductive output was generally negative regardless of germination strategy. Warming significantly reduced the number of inflorescences produced and the number of viable infructescences per plant across strategies (Fig. 1, Fig. 3a and b, Table 1). Overall, there was also a significant reduction in the total number of plants flowering (Fig. 4, Table 1) under warmed conditions.

Individual seed mass varied among germination strategies with seed of the immediate strategy being lightest and staggered being heaviest, but warming did not result in any change in seed mass (Fig. 1, Fig. 3c, Table 1). Compared to the field collected seed, mass of the seed produced in the soil warming experiment was up to 1.2 mg per seed heavier than the seed collected from the field (parent), except for those exhibiting an immediate germination strategy, where seed mass between field and experiment was constant (Fig. 3c).

## Phenology

The number of days to first flowering significantly varied with germination strategy and was not affected by soil warming (Fig. 1, Fig. 4, Table 1). Although warming did not affect flowering time, it accelerated time to seed maturity and reduced the overall duration of seed production. Immediate germination strategy plants flowered 50 days earlier, and the seeds were relatively slower to mature compared to the three other strategies (Fig. 4, Table 1). Consistent with their early flowering date, absolute timing of seed maturity of immediate strategy was earlier compared to the other strategies (Fig. 4, Table 1). Finally, the plants senesced earlier in warmer soil than in ambient soil (Fig. 1, Fig. 4, Table 1). At the end of the experiment, a greater proportion of plants survived in the ambient than warmed conditions for all germination strategies except the postponed strategy, in which mortality was approximately 95% regardless of growth temperature (Fig. 4, Table 1).

## Transgenerational effects of warming

Final germination of F1 seeds across germination strategies and soil temperature treatments was >90%, indicating that all seeds produced were healthy and viable regardless of maternal soil temperature. Germination temperature, in contrast, affected both timing and the fraction of seed that were dormant (Fig. 3d). Warmer germination temperature led to longer time to reach 50% germination and reduced germination of the non-dormant seed fraction regardless of the germination strategy and maternal warming (Table 2, Table S3). The absence of an interaction effect between germination temperature and maternal warming indicates that maternal soil temperatures did not affect the response to germination temperatures, such that seeds produced from plants in warm soil did not do better at higher germination temperatures than seeds produced from plants in ambient soil. Across generations, germination strategies were not only conserved across strategies (Fig. 3d), but also within strategy, i.e. plants of staggered strategy produced seeds that had a staggered germination strategy regardless of whether the maternal plant was an autumn or spring germinant (Fig. S2). In addition, the proportion of dormant and non-dormant seeds within staggered populations was relatively constant (Fig. S2).

## Discussion

This study examined the effect of soil warming on a whole-of-life array of traits (vegetative growth, reproductive output and phenological response traits including senescence), as well as the germination traits of the next generation, for populations of *O. eriopoda* with four different germination strategies. Our findings show that responses to soil warming and germination strategy vary among traits and as a function of germination strategy. Since we followed the whole life cycle, we were able to observe that the effect of soil warming on traits changed through ontogeny and was mostly apparent in the respective active growth stages of the plant life cycle, demonstrating that care must be taken in extrapolating from responses of a given trait at any point in time to a whole-of-life conclusion. The diversity of these responses to warming throughout the life cycle, and among germination strategies within a species, highlight the complexity of linkages between the maternal and offspring environment and make evident that without a whole-of-life perspective we will struggle to predict impacts of global change on species, never mind to understand the mechanisms underlying those impacts. Here we interpret these elements in the context of impacts of warming on persistence of *O. eriopoda* and other alpine species in a novel future climate.

Under rapid climate change, plasticity in phenology and reproductive traits is likely to have strong fitness consequences (Kozłowski, 1992, Stinson, 2004), and it is possible that different germination strategies will have different selective advantages (Hoyle *et al.*, 2014, Willis *et al.*, 2014). We found that variation in reproductive phenology (Fig. 4) and seed mass (Fig. 3) were inherent to the germination strategy, whereas adult vegetative traits and reproductive outputs were more strongly affected by warming than germination strategy. The ability of this species to adjust reproductive phenology by shortening seed maturation while maintaining seed quality (seed mass and viability) may be advantageous for persistence under in changing climate (Bonser, 2013, Visser and Both, 2005, Willis *et al.*, 2008); even if the total seed number is reduced, the seeds are spread through the season. Although warming substantially reduced lifespan, it was apparent that *O. eriopoda* individuals could complete their life cycle and produce healthy, full-size seed with adequate reserves for early establishment success. Nearly all of the seeds produced were viable regardless of germination temperature, maternal conditions or germination strategy indicating potential to maintain population regeneration.

The interactions of soil warming and germination strategy were mainly evident for seedlings or early vegetative traits and not vegetative traits during the transition to reproductive stage; this corroborates the finding of a previous study by Hoyle *et al.* (2015) that germination strategy of Australian alpine plants does not correlate with adult vegetative traits. In particular, populations of the immediate germination strategy (mainly occupy lower <1520 m elevation sites) exhibited greater plasticity in early leaf increment in response to warming compared to the staggered, postponed and postponed deep strategies (elevations ranging from 1600 m to 2200 m, Supplemental Table 1). Previous research on *Wahlenbergia ceracea*, an alpine herb that shares the same habitat with *O. eriopoda*, also found individuals from higher elevations were less plastic and less likely to express adaptive plasticity in growth response to warming (Nicotra *et al.*, 2015). The positive response of *O. eriopoda* vegetative traits to warming that was mainly pronounced during earlier ontogeny, however, did not lead to greater growth accumulation and earlier reproductive timing (flowering). The results indicate that there might be an internal constraint for vegetative growth and maintenance (Starr *et al.*, 2000) and thus individuals with an immediate germination strategy grew and reached the reproductive stage more quickly but also died earlier than individuals displaying the other strategies.

By germinating the seeds produced in the warming experiment we confirmed that the source of variation in timing of germination in the staggered populations lies within individual plants, indicating a potential bet-hedging strategy (Starrfelt and Kokko, 2012), which has not been verified in this species before (Hoyle *et al.*, 2015, Satyanti *et al.*, 2019). Interestingly, individual plants produced both non-dormant and dormant seed of varying proportions (Fig. S3). This leads to asynchronous germination within the population and can reduce the risk against recruitment failure (Brown and Venable, 1986, Simons Andrew, 2009, Stevens *et al.*, 2014, Venable and Lawlor, 1980). Should variation in snowmelt patterns compromise recruitment in either season, populations with a staggered strategy could be advantaged and this raises a question about how germination strategy is established and controlled in the species; its high variability indicates it is highly labile on some timeframe.

We examined the transgenerational effect of different seed development (maternal) conditions on germination traits since this has been proposed as a mechanism that may help species to tolerate future climates (Herman and Sultan, 2011). Given how variable germination strategy is we hypothesized that it may be highly plastic and reflect developmental conditions. But, contrary to expectation, we found little evidence of phenotypic plasticity in seed dormancy, i.e. germination strategy across *O. eriopoda* populations was constant regardless of seed development temperature. The warming impact was imposed when the plants were 18–20 weeks old, not the earliest seedling stage, but given that warming was imposed before the transition to reproductive meristems, this delay seems unlikely to have impeded the response. Our results suggest that seed dormancy variation is not highly dependent on seed maturation environment. Seed development temperatures have been shown to control seed dormancy induction and cycling in other species (Bernareggi *et al.*, 2016, Donohue *et al.*, 2005, Footitt and Finch-Savage, 2017, Steadman *et al.*, 2004), but we do not know the relative contributions of air or soil temperature. Further, the degree of seed dormancy, the important determinant of germination strategy and dormancy cycling may indeed depend on variation in and not just the mean of temperature (Satyanti *et al.*, 2019, Topham *et al.*, 2017).

Alternatively, it may be the case that germination strategy is plastic but has a ‘half-life’. It is possible that more than one cycle of warming is required to elicit large-scale change the dormancy degree or fraction of non-dormant seed of the respective population. We found warming elicited a small increase in dormancy across all germination strategies, but the immediate populations still had nearly 90% non-dormant seed. Previous studies suggest that the warming effects on seed dormancy and germination traits may be gradual rather than instant, just like phenological trait responses to warming (Franks *et al.*, 2007, Hoffmann *et al.*, 2010). Such a delay effect may indicate that the mechanism underlying this shift is epigenetic. Dormancy patterns have been shown to be under epigenetic control in other systems (Nonogaki, 2017, Richards *et al.*, 2017), but further research would be needed to demonstrate thathere.

Diversification of germination strategies across populations, as exhibited by *O. eriopoda*, could still be an advantage that assists species’ persistence (Cochrane *et al.*, 2015b). The potential for plants to respond to warming not only plastically but also as a function of genetic (or epigenetic) variation within the species demonstrates that species’ response to warming will often be manifested as a combination of rapid plastic responses and long-term evolutionary responses (Valdés *et al.*, 2019). Considering the spatial and temporal heterogeneity of the alpine environment it is intuitive to expect variations in seed trait, particularly in dormancy, and hence we suggest that variation in germination phenology may be quite common within alpine species (Venable and Brown, 1988). Very few studies to date have documented intraspecific variation in seed dormancy and germination strategy as we have here. We have shown that *O. eriopoda* has populations with non-dormant seed (immediate germination strategy) as well as dormant seed in various proportion (staggered and postponed strategy) and that some populations have deeply dormant seeds (postponed-deep strategy). How common this is among alpine species and how that affects their potential resilience under future warming remain to beseen.

An important implication of the results of our study is that predicting species’ responses and fate under global warming as either positive, negative or neutral could be a gross oversimplification when such assessments consider only one or few populations or are based only on a limited number of life stages or traits (Saatkamp *et al.*, 2018). The effects of warming vary not only among populations and individuals but as a function of ontogeny and hence, when assessing response to climate change at both species and community levels, within-species variations in germination strategy should be considered as important as between-species variation, and impacts must be assessed on a whole-of-life scale, not just at a single life stage. For *O. eriopoda*, warming will have some potentially positive effects (early growth rates) and some negative effects (reduced reproductive output). But ultimately, the effect of warming will depend on how those effects play out in the field: early establishment and accelerated trajectory to seed maturity may offset the tradeoff with overall seed production. Small differences among germination strategies likewise may cascade to larger effects, shifting their representation across the landscape, with important implications for persistence. Thus, we conclude that to understand the response of wild species to warming takes a whole-of-life perspective and attention to ecologically significant patterns of within-species variation.

The outcomes of this study are meaningful for conservation and management of Australian alpine species as they can inform predictions of alpine plant responses to changing climate. Such information can help prioritize species for *in situ* management or form the basis of *ex situ* conservation and restoration actions. For example, these results would lead to better seed propagation plans for this species and an improved collection strategy for future seed banking to ensure representation of populations and germination strategies in *ex situ* collections. *O. eriopoda* populations are well represented across Australian seed banks and this will not only be meaningful for safeguarding the species but also making in-depth research on germination ecology possible. For many species, however, this is not the case. This study demonstrates the importance of collecting and documenting the seed biology of a thorough representation of populations across species distribution for the purposes of conservation seed banking.

## Authors’ contributions

A.S., A.N. and L.G. conceived the ideas, designed the methodology and led the writing of the manuscript. A.S., T.L. and M.T. collected the data. A.S. and T.N. analysed the data. All authors contributed substantially to the drafts and gave final approval for publication.

## Data availability

Data will be archived at AusTraits on publication.

## Supplementary Material

Satyanti_Supporting_Information_ConPhys_Feb_21_coab023
