# Supplementary material for: Predicting effects of warming requires a whole-of-life cycle perspective: a case study in the alpine herb Oreomyrrhis eriopoda
Source: Conserv Physiol. 2021 Apr 28;9(1):coab023. doi: 10.1093/conphys/coab023 (PMC8084022; doi:10.1093/conphys/coab023)
Supplement: Satyanti_Supporting_Information_ConPhys_Feb_21_coab023 [file satyanti_supporting_information_conphys_feb_21_coab023.docx]

**Supporting Information**

**Appendix 1**

**Germination and seedling transplant methods**

Seeds from each population were germinated in three replicates of 25 seeds per population and were placed on 1% water-agar in sealed petri dishes in a germination chamber (Model TRIL-120-1-VW/ BMS, Serial 30455, Thermoline Scientific, NSW, Australia). Dishes for populations with the postponed and postponed deep strategy were exposed to at 25/ 15 °C, 12 hours light/ 12 hours dark, for 9 weeks and then were moved to 5 °C constant temperature and 12 hours light/ 12 hours dark for 8 weeks before being returned to 25/ 15 °C, 12 hours light/ 12 hours dark, for 27 weeks. For populations that exhibit immediate or staggered strategy (Nam1, Nam2, Kos12, Kos21, and Kos22, Tas8, and Tas9) an additional set of dishes was germinated after the first set had been brought out of ‘winter’ conditions so that germination of early (autumn) and late (spring) seedlings coincided and that seedling age was relatively equal regardless of germination strategy. We did not have enough seeds for staggered populations of Kos3 and Kos6, and hence could only use the late (spring) seedlings for Kos3 and Kos6.

All germinating seeds were transplanted to potting mix when the radicle was just over 1 cm (1 – 2 weeks). Seedlings were grown in 4 × 4 × 10 cm pots (T40S, Garden City Plastics, NSW, Australia), one seedling per pot, with a mixture of Martins mix potting-soil (Martins Fertilizer, NSW, Australia) plus 10% steamed river sand and c. 4 gram/ pot of slow release fertilizer (Osmocote Exact Patterned Release Fertilizer Standard Blue 15% N: 4% P: 7.5% K, Scotts International BV, Heerlen, The Netherlands). The glasshouse environment was kept at 25/15 °C (day/night) with a natural spring/ summer photoperiod of Canberra, ACT, Australia. Seedling position was shuffled within and across glasshouse benches every 1-2 weeks. Seedlings were grown until they were 18 – 20 weeks old to ensure that plants across all populations were large enough to withstand transplant stress and thus to avoid confounding soil warming effects. Immediately before we started the warming experiment, the seedlings were re-potted with the same mixture and fertilizer as above into larger 7 x 7 x 20 cm pots (T70VINK Garden City Plastics, NSW, Australia).

**
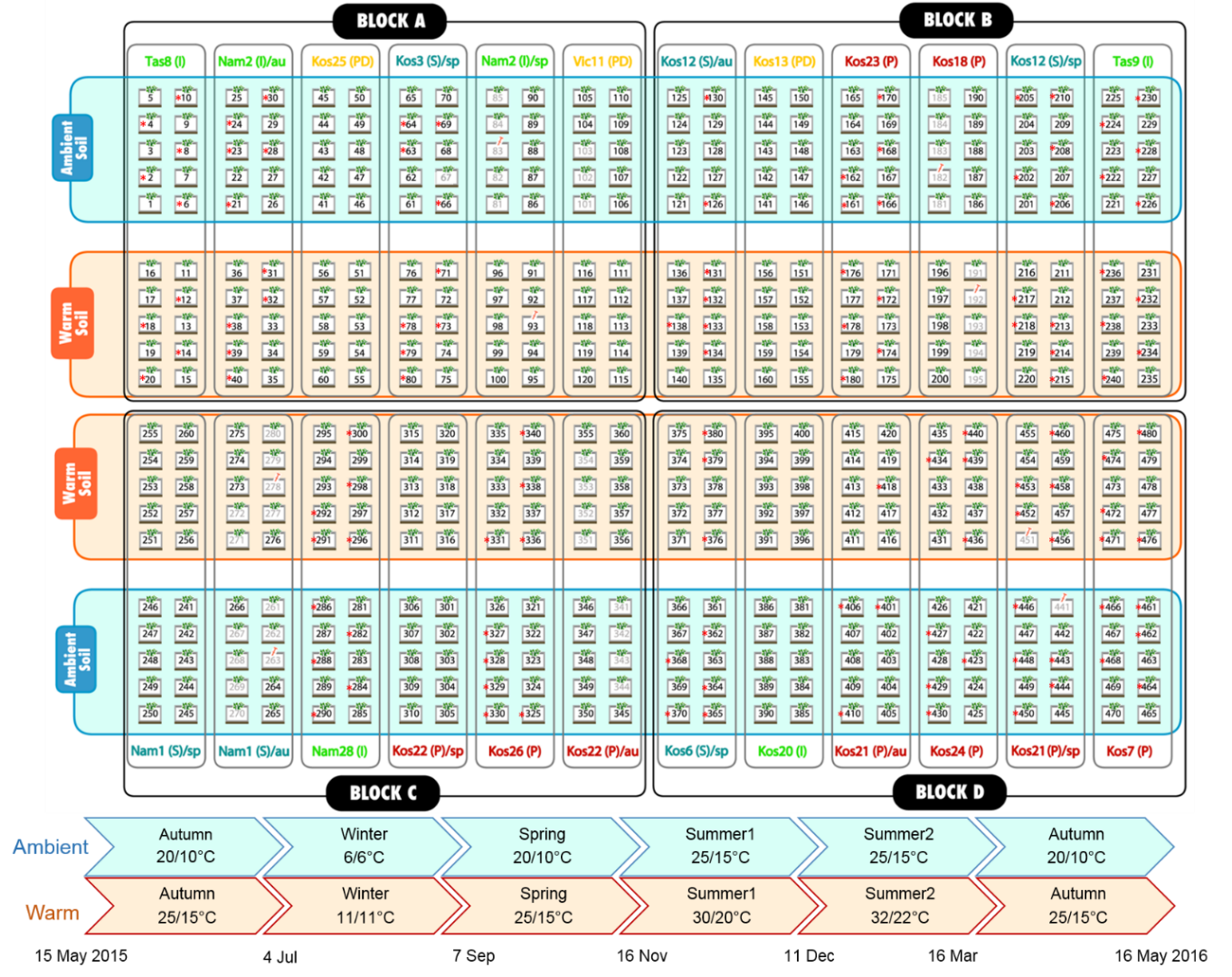
**

**Supplementary Figure 1.** The detailed population and germination strategy allocations across blocks with the targeted temperature regime and the seasons. Population names are coloured based on germination strategy and followed by seedling germination season if two germination timings occurred within a population. Germination strategies were: Immediate (I), Staggered (S), Postponed (P), and Postponed-deep (PD); autumn (early) germinating seed was coded as ‘(/au)’ and spring (late) germinating seed as ‘(/sp)’. Temperature regime is shown along the bottom. Note that there were two stages of summer temperature for the warmed treatment, thus we have Summer1 and Summer2 shown separately. Where plant availability was low, extra pots were placed into the experiment to ensure equivalent neighbour effects for all pots. Those extras were not part of the analysis and the pots are shown with grey labels. Red asterisks indicate individuals from which seed was sourced for the trans-generational experiment. Each pot contained one plant. Pots with orange pin (without plant; pot number 83, 93, 182, 192, 263, 278, 441 and 451) were the pots allocated for i-Buttons (3 i-Buttons below the soil and 1 i-Button above the soil) in each block.


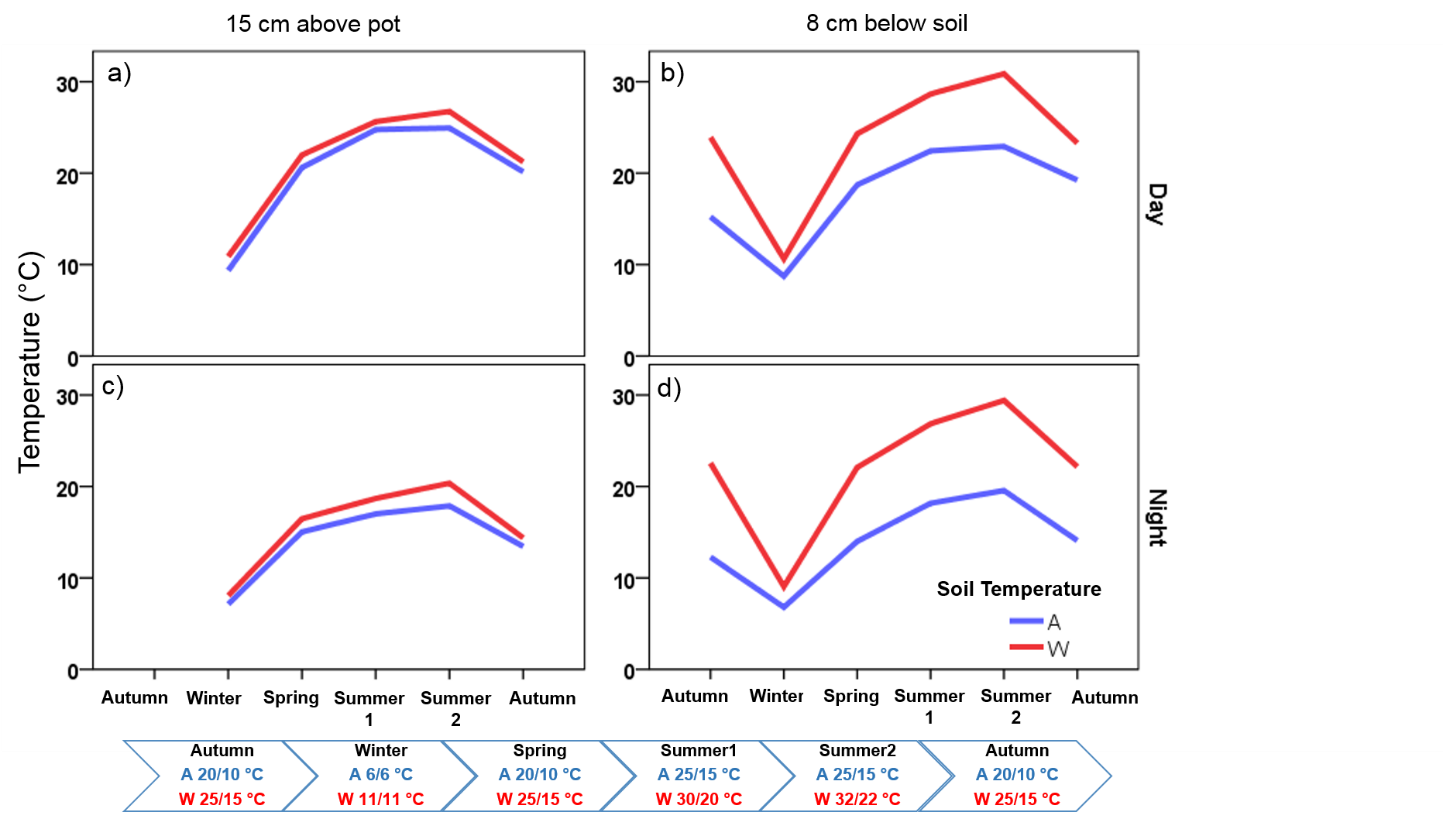


**Supplementary Figure 2:** The realised mean temperature in ambient and warm conditions during the day at a) plant level 15 cm above the pot, and b) 8 cm below the soil surface; and during the night, c) plant level 15 cm above the pot, and d) 8 cm below the soil surface. At each season, warm conditions were significantly warmer than ambient (p < 0.001, ANOVA). Note: data loggers for plant level temperatures were only started in winter. The boxes along the bottom of the figure indicate the target soil temperatures for ambient (A) and warm (W) treatment for each season. Summer1 and Summer2 indicate the two steps of summer conditions for the warm treatment.


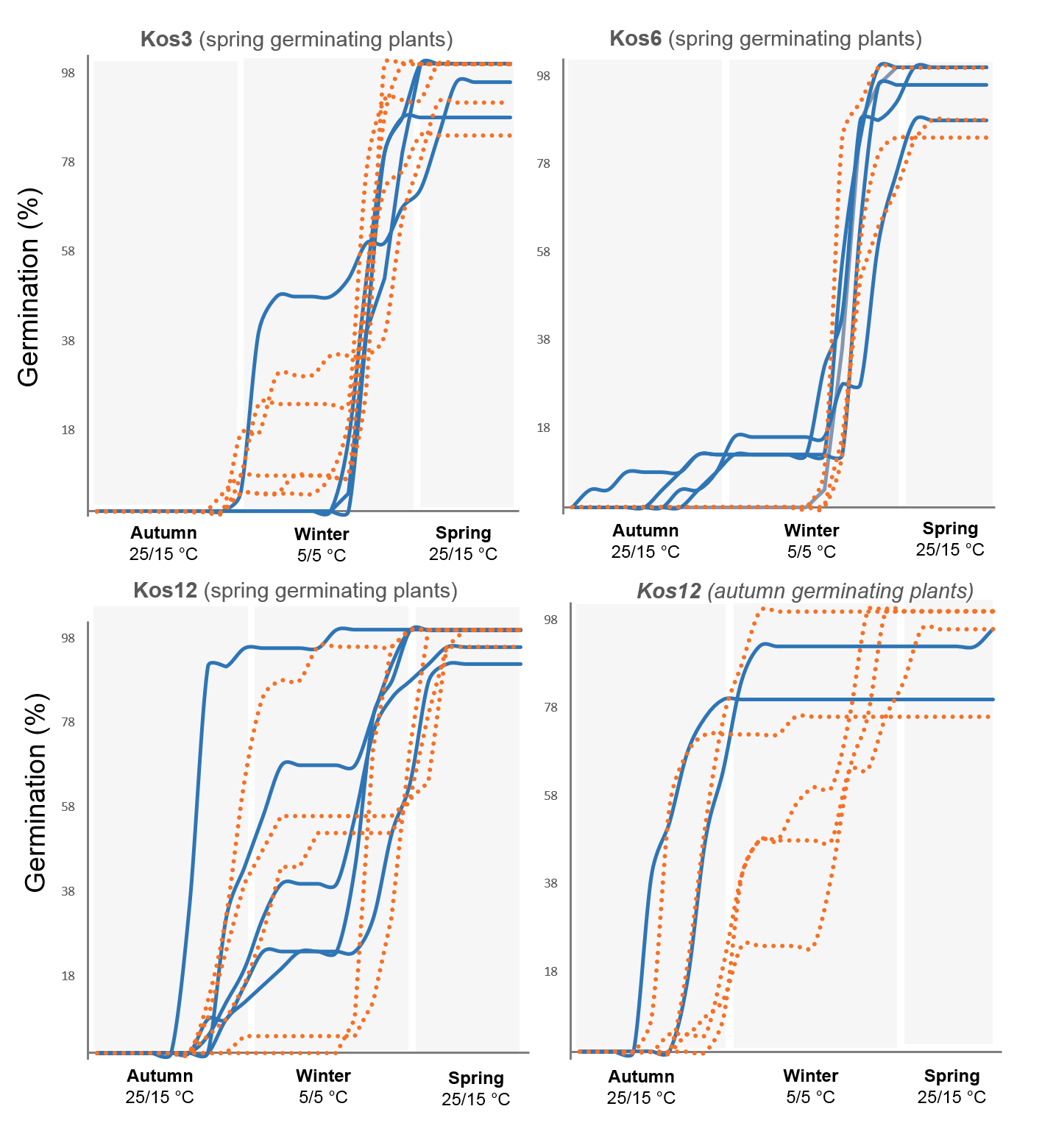


**Supplementary Figure 3**. Offspring germination of the staggered strategy under ambient germination temperature over time (indicated by x-axis). Lines indicate which individual (mother plant) produced the seeds; each line represents 100 seeds. Line colour and style indicate maternal soil temperature – solid blue for ambient soils and dashed orange for warmer soil temperatures.

| Table S1. The distribution of population and germination strategies (GS) across the blocks: Immediate (I), Staggered (S), Postponed (P), and Postponed-deep (PD). Population name reflects the collection sites: Namadgi (Nam), Kosciuszko (Kos), Victoria (Vic), and Tasmania (Tas). Seeds for each population were bulk sampled from multiple plants. There was imbalance in the distribution of germination strategies across blocks, but this was not so extensive as to be an impediment to statistical analyses. Accessions with grey font are those re-determined as other *Oreomyrrhis* species, new species ID in bracket, and thus were excluded in the analyses. | | | | | | | | |
| --- | --- | --- | --- | --- | --- | --- | --- | --- |
| **Block** | **GS** | **Population** | **Accession** | **Lat.** | **Long.** | **Elevation** | **Collection date** | **Locality** |
| **A** | I | Nam2 (*O. ciliata*) | CANB 866345 | -35.568 | 148.7844 | 1647 | 19-Feb-13 | Namadgi NP. Snowy Flat, southern end; Mt Franklin Road, c. 5 km S of locked gate near Mt Ginini. |
|  | I | Tas8 | TSCC 0009097 | -42.151 | 146.4604 | 660 | 16-Feb-09 | Tasmania, Nive Plains. Lyell Highway, 500 m NW of Nive River Bridge |
|  | S | Kos3 | NSW 618325 | -36.371 | 148.4761 | 1608 | 10-Feb-04 | Kosciuszko NP. Rainbow Lake Track, off Kosciuszko Road between Perisher Valley and Guthega turn-off; Kosciuszko NP Southern Tablelands. |
|  | P | N/A | N/A | N/A | N/A | N/A | N/A | N/A |
|  | PD | Kos25 | CANB 792151 | -36.429 | 148.3586 | 1744 | 3-Feb-10 | Kosciuszko NP. S side of Spencers Creek bridge; roadside and water side. |
|  | PD | Vic11 | MEL 2338193 | -37.06 | 147.092 | 1632 | 25-Jan-11 | Alpine NP, Victoria. The Dargo High Plains Road about 16.1 km from the Alpine Road. |
| **B** | I | Tas9 | TSCC 0005951 | -41.958 | 146.6786 | 1055 | 24-Jan-08 | Tasmania, Great Lake. Cameroon lagoon, small wetland on west side of Lake Highway; 7 km S of Liawenee. Subalpine health, associated with *Ozothamnus* spp; *Poa* spp. |
|  | S | Kos12 | CANB 783437 | -36.427 | 148.3677 | 1743 | 24-Feb-09 | Kosciuszko NP. Fens along Spencers Creek – 790 m NE from the Spencers Creek bridge on Kosciuszko Road. |
|  | P | Kos18 | CANB 783604 | -36.434 | 148.3066 | 1805 | 5-Feb-09 | Kosciuszko NP. The Main Range, about 1.4 km SW from the walking track to Blue Lake crosses the Snowy River. |
|  | P | Kos23 | CANB 747602 | -36.463 | 148.2677 | 2079 | 14-Feb-07 | Kosciuszko NP. Bog’s margin, 800 m ~NE from Lake Cootapatamba. |
|  | PD | Kos13 | CANB 807987 | -36.429 | 148.3586 | 1702 | 28-Feb-12 | Kosciuszko NP. The Kosciuszko Road at the Spencers Creek bridge, c. 50 m W of bridge on S side of road. |
| **C** | I | Nam28 | CBG 9604821 | -35.635 | 148.78 | 1520 | 5-Mar-96 | Namadgi NP. Leura Gap, ca 3 km direct NNW of Bimberi Peak. |
|  | S | Nam1 (*O. ciliat*a) | CANB 813617 | -35.522 | 148.7719 | 1621 | 17-Jan-13 | Namadgi NP. Ginini West *Sphagnum* peat bog southern part, 0.5 km W from the Mt Ginini carpark on the Mt Franklin Road towards Bulls Head. |
|  | P | Kos22 | CANB 770116 | -36.452 | 148.2747 | 2078 | 5-Mar-08 | Kosciuszko NP. Kosciuszko summit road c. 1 km from Seaman's Hut. |
|  | PD | N/A | N/A | N/A | N/A | N/A | N/A | N/A |
|  | P | Kos26 | CANB 792159 | -36.416 | 148.3111 | 1936 | 4-Feb-10 | Kosciuszko NP. N side of path, overlooking Headley Tarn. |
| **D** | I | Kos20 (*O. argentea*) | CANB 749000 | -35.826 | 148.4927 | 1399 | 2-Mar-07 | Kosciuszko NP. Roadside at Old Kiandra Goldfields, 3 km N of turn-off to Cabramurra and Khancoban. |
|  | S | Kos6 | NSW 4154437 | -36.45 | 148.3167 | 1800 | 1-Mar-88 | Kosciuszko NP. 1 km along the summit walking track to Mt Stillwell. |
|  | P | Kos7 | NSW 617524 | -36.491 | 148.285 | 1960 | 24-Feb-04 | Kosciuszko NP. Kosciuszko NP Southern Tablelands, approximately 400 m NW of the top of Crackenback chairlift, on walking track to Mt Kosciuszko. |
|  | P | Kos21 | CANB 748561 | -36.439 | 148.2702 | 2011 | 7-Mar-07 | Kosciuszko NP. 2 km N of Mt Kosciuszko summit, on Main Range track. |
|  | P | Kos24 | CANB 792219 | -36.453 | 148.265 | 2159 | 30-Mar-10 | Kosciuszko NP. Lake Albina Track. |
|  | PD | N/A | N/A | N/A | N/A | N/A | N/A | N/A |

**Supplementary Table 2**. Significance of the germination strategy (GS) and warming (SoilT) treatments on the vegetative and reproductive traits, and on phenology. Generalized Linear Mixed model and Linear Mixed Models were used to analyse the data with fixed term set as GS×SoilT. The random term was population nested in block (block/pop). Plant area and aboveground biomass were transformed using natural logarithms. In the direction column, germination strategies are noted as Immediate (I), Staggered (S), Postponed (P), and Postponed-deep (PD) and SoilT as Ambient (A) and Warm (W).

| **Response** | **Fixed term** | **Wald statistic** | **n.d.f.** | **F statistic** | **d.d.f.** | **F pr** | **Direction** |
| --- | --- | --- | --- | --- | --- | --- | --- |
| **Start of the experiment^a^** |  |  |  |  |  |  |  |
| Leaf number | GS | 63.48 | 3 | 21.16 | 11.7 | **<0.001** | **(S, P)<PD<I** |
| Longest leaf | GS | 2.29 | 3 | 0.76 | 12 | 0.537 |  |
|  |  |  |  |  |  |  |  |
| **Early vegetative stage** |  |  |  |  |  |  |  |
| Leaf number ^b^ ‡ | GS | 10.02 | 3 | 3.29 | 10.7 | 0.063 |  |
|  | SoilT | 64.91 | 1 | 64.91 | 301.1 | **<0.001** | **A<W** |
|  | GS×SoilT | 21.55 | 3 | 7.18 | 301.1 | **<0.001** | **I(A<W), PD(A<W)** |
| Leaf increment rate (leaf per day) ^b^ ‡ | GS | 9.88 | 3 | 3.24 | 11.2 | 0.063 |  |
|  | SoilT | 58.25 | 1 | 58.25 | 301.2 | **<0.001** | **A<W** |
|  | GS×SoilT | 48.58 | 3 | 16.19 | 300.7 | **<0.001** | **I(A<W), PD(A<W)** |
| Longest leaf † | GS | 80.77 | 3 | 26.89 | 230.5 | **<0.001** | **I<S<PD<P** |
|  | SoilT | 0.24 | 1 | 0.24 | 311 | 0.626 |  |
|  | GS×SoilT | 8.23 | 3 | 2.74 | 311 | **0.043** | **I(A>W)** |
| Specific Leaf Area ^b^ ‡ | GS | 12.4 | 3 | 4.12 | 9.3 | **0.041** | **P<PD<S<I** |
|  | SoilT | 28.16 | 1 | 28.16 | 300.2 | **<0.001** | **A<W** |
|  | GS×SoilT | 0.57 | 3 | 0.19 | 300.2 | 0.903 |  |
|  |  |  |  |  |  |  |  |
| **Transition to Reproductive Stage** | |  |  |  |  |  |  |
| Leaf number ^b^ ‡ | GS | 5.39 | 3 | 1.78 | 11.3 | 0.208 |  |
|  | SoilT | 0.83 | 1 | 0.83 | 112.3 | 0.363 |  |
|  | GS×SoilT | 2.21 | 3 | 0.74 | 112.3 | 0.533 |  |
| Leaf increment rate (leaf per day) ^b^ ‡ | GS | 3.11 | 3 | 1.02 | 11.9 | 0.417 |  |
|  | SoilT | 8.44 | 1 | 8.44 | 111.2 | **0.004** | **A>W** |
|  | GS×SoilT | 4.99 | 3 | 1.66 | 111 | 0.179 |  |
| Longest leaf | GS | 11.91 | 3 | 3.9 | 10.2 | **0.043** | **I<(S, P, PD)** |
|  | SoilT | 0.07 | 1 | 0.07 | 112.5 | 0.793 |  |
|  | GS×SoilT | 0.28 | 3 | 0.09 | 112.4 | 0.963 |  |
| Canopy area ^b^ ‡ | GS | 2.82 | 3 | 0.93 | 11 | 0.458 |  |
|  | SoilT | 0.02 | 1 | 0.02 | 110.2 | 0.875 |  |
|  | GS×SoilT | 3.44 | 3 | 1.15 | 110.6 | 0.333 |  |
|  |  |  |  |  |  |  |  |
| **Reproductive stage** |  |  |  |  |  |  |  |
| Proportion of flowering plant ^b^ † | GS | 5.06 | 3 | 1.66 | 49 | 0.189 |  |
|  | SoilT | 4.9 | 1 | 4.9 | 311 | **0.028** | **A>W** |
|  | GS×SoilT | 6.85 | 3 | 2.28 | 311 | 0.079 |  |
| Total number of inflorescence ^b^ † | GS | 5.48 | 3 | 1.83 | 232.3 | 0.143 |  |
|  | SoilT | 26.13 | 1 | 26.13 | 249.3 | **<0.001** | **A>W** |
|  | GS×SoilT | 6.47 | 3 | 2.16 | 249.2 | 0.094 |  |
| Total number of infructescence ^b^ † | GS | 4.04 | 3 | 1.35 | 246.2 | 0.260 |  |
|  | SoilT | 12.33 | 1 | 12.33 | 249.1 | **<0.001** | **A>W** |
|  | GS×SoilT | 6.47 | 3 | 2.16 | 249.1 | 0.094 |  |
| Single seed mass ^b^ | GS | 141.43 | 3 | 46.71 | 10.5 | **<0.001** | **I<(S, PD)<P** |
|  | SoilT | 0.4 | 1 | 0.4 | 177.9 | 0.527 |  |
|  | GS×SoilT | 5.03 | 3 | 1.68 | 177.7 | 0.174 |  |
| Proportion of infructescence to inflorescence † | GS | 26.45 | 3 | 8.64 | 48.8 | **<0.001** | **I>(S, P, PD)** |
|  | SoilT | 0.28 | 1 | 0.28 | 251.3 | 0.597 |  |
|  | GS×SoilT | 7.63 | 3 | 2.54 | 250.3 | 0.057 |  |
| Aboveground biomass final ^b^ ‡ | GS | 1.55 | 3 | 0.5 | 7.5 | 0.695 |  |
|  | SoilT | 5.54 | 1 | 5.54 | 274.1 | **0.019** | **A>W** |
|  | GS×SoilT | 2.96 | 3 | 0.99 | 273.2 | 0.399 |  |
| Proportion plants surviving ^b^ | GS | 5.56 | 3 | 1.85 | 8.4 | 0.214 |  |
|  | SoilT | 7.09 | 1 | 7.09 | 307.8 | **0.008** | **A>W** |
|  | GS×SoilT | 11.91 | 3 | 3.97 | 305.6 | **0.008** | **I(A>W), S(A>W), PD(A>W)** |
|  |  |  |  |  |  |  |  |
| **Phenology** |  |  |  |  |  |  |  |
| Days to first flower ^b^ | GS | 79.55 | 3 | 26.5 | 11.6 | **<0.001** | **I<(S, P, PD)** |
|  | SoilT | 0.21 | 1 | 0.21 | 258.2 | 0.645 |  |
|  | GS×SoilT | 4.26 | 3 | 1.42 | 258 | 0.238 |  |
| Days to seed maturity from flowering ^b^ | GS | 43.18 | 3 | 14.21 | 10 | **<0.001** | **I>(S, P, PD)** |
|  | SoilT | 10.29 | 1 | 10.29 | 248 | **0.002** | **A>W** |
|  | GS×SoilT | 5.55 | 3 | 1.85 | 248.4 | 0.139 |  |
| Days to seed maturity from planting | GS | 17.27 | 3 | 5.64 | 10.2 | **0.015** | **I<(S, P, PD)** |
|  | SoilT | 6.24 | 1 | 6.24 | 246.9 | **0.013** | **A>W** |
|  | GS×SoilT | 3.56 | 3 | 1.19 | 247 | 0.316 |  |
| Duration of seed production ^b^ † | GS | 56.57 | 3 | 18.86 | 257 | **<0.001** | **I>(S, P, PD)** |
|  | SoilT | 7.48 | 1 | 7.48 | 257 | **0.007** | **A>W** |
|  | GS×SoilT | 3.58 | 3 | 1.19 | 257 | 0.312 |  |
| Days to senesce from last seed production | GS | 2.95 | 3 | 0.98 | 11.2 | 0.438 |  |
|  | SoilT | 1.9 | 1 | 1.9 | 220.6 | 0.169 |  |
|  | GS×SoilT | 3.31 | 3 | 1.1 | 221.3 | 0.349 |  |
| Days to senesce from planting ^b^ | GS | 17.27 | 3 | 5.64 | 10.2 | **0.015** | **I<P<PD<S** |
|  | SoilT | 6.24 | 1 | 6.24 | 246.9 | **0.013** | **A>W** |
|  | GS×SoilT | 3.56 | 3 | 1.19 | 247 | 0.316 |  |

a indicates that the fixed factor was GS and random was population as the variables were measured before the experiment started.

b indicates the result is also presented in Table 1.

‡ initial number of leaves (at day 0) was added as a covariate.

† random factor was block

**Supplementary Table 3**. Transgenerational effects on germination traits. The significance of germination strategy (GS), soil temperature (SoilT), and germination temperature (GermT). The analyses were performed using mixed models with GS×SoilT×GermT as fixed effects and population and individual plant nested in shelf as random factors. Direction column as for Supp. Table 2.

| **Response** | **Fixed term** | **Wald statistic** | **n.d.f.** | **F statistic** | **d.d.f.** | **F pr** | **Direction** |
| --- | --- | --- | --- | --- | --- | --- | --- |
| Final germination | GS | 4.84 | 2 | 2.42 | 196.4 | 0.092 |  |
|  | SoilT | 0.04 | 1 | 0.04 | 211.9 | 0.849 |  |
|  | GermT | 3.38 | 1 | 3.38 | 211.3 | 0.067 |  |
|  | GS×SoilT | 1.01 | 2 | 0.51 | 212.2 | 0.603 |  |
|  | GS×GermT | 1.6 | 2 | 0.8 | 211.3 | 0.45 |  |
|  | SoilT×GermT | 0.91 | 1 | 0.91 | 211.3 | 0.342 |  |
|  | GS×SoilT×GermT | 1.09 | 2 | 0.55 | 211.3 | 0.58 |  |
| Non-dormant fraction | GS | 16.66 | 2 | 8.33 | 7.6 | **0.012** | **I>S>P** |
|  | SoilT | 0.89 | 1 | 0.89 | 204.1 | 0.347 |  |
|  | GermT | 24.51 | 1 | 24.51 | 202.8 | **<0.001** | **A>W** |
|  | GS×SoilT | 2.31 | 2 | 1.15 | 203.9 | 0.318 |  |
|  | GS×GermT | 4.11 | 2 | 2.05 | 202.6 | 0.131 |  |
|  | SoilT×GermT | 1.04 | 1 | 1.04 | 202.3 | 0.308 |  |
|  | GS×SoilT×GermT | 1.72 | 2 | 0.86 | 202.3 | 0.425 |  |
| Time to reach 50% germination | GS | 78.95 | 2 | 39.47 | 8.1 | **<0.001** | **I<(S=P)** |
|  | SoilT | 0.55 | 1 | 0.55 | 205.3 | 0.461 |  |
|  | GermT | 19.52 | 1 | 19.52 | 203.4 | **<0.001** | **W>A** |
|  | GS×SoilT | 2.99 | 2 | 1.49 | 204.5 | 0.227 |  |
|  | GS×GermT | 3.33 | 2 | 1.66 | 203.4 | 0.192 |  |
|  | SoilT×GermT | 2.26 | 1 | 2.26 | 203.4 | 0.135 |  |
|  | GS×SoilT×GermT | 0.57 | 2 | 0.28 | 203.4 | 0.753 |  |
